# Supplementary material for: Crawling and Gliding: A Computational Model for Shape-Driven Cell Migration
Source: PLoS Comput Biol. 2015 Oct 21;11(10):e1004280. doi: 10.1371/journal.pcbi.1004280 (PMC4619082; doi:10.1371/journal.pcbi.1004280)
Supplement: S1 Code — (ZIP) [file pcbi.1004280.s012.zip › release/tst/doc/html/random_8h_source.html]

Tissue Simulation Toolkit: random.h Source File


Tissue Simulation Toolkit
 0.1.4.1


- Main Page
- Namespaces
- Classes
- Files

- File List
- File Members

random.h

Go to the documentation of this file.

1 /\*

2

3 Copyright 1996-2006 Roeland Merks

4

5 This file is part of Tissue Simulation Toolkit.

6

7 Tissue Simulation Toolkit is free software; you can redistribute

8 it and/or modify it under the terms of the GNU General Public

9 License as published by the Free Software Foundation; either

10 version 2 of the License, or (at your option) any later version.

11

12 Tissue Simulation Toolkit is distributed in the hope that it will

13 be useful, but WITHOUT ANY WARRANTY; without even the implied

14 warranty of MERCHANTABILITY or FITNESS FOR A PARTICULAR PURPOSE.

15 See the GNU General Public License for more details.

16

17 You should have received a copy of the GNU General Public License

18 along with Tissue Simulation Toolkit; if not, write to the Free

19 Software Foundation, Inc., 51 Franklin St, Fifth Floor, Boston, MA

20 02110-1301 USA

21

22 \*/

23 #define MBIG 1000000000

24 #define MSEED 161803398

25 #define MZ 0

26 #define FAC (1.0/MBIG)

27

28 double RANDOM();

29 int Seed(int seed);

30 long RandomNumber(long max);

31 void AskSeed();

32 int Randomize(void);

33

34

35

36

37

38

39

max

#define max(x, y)

**Definition:** conrec.cpp:10

Randomize

int Randomize(void)

**Definition:** random.cpp:124

RandomNumber

long RandomNumber(long max)

**Definition:** random.cpp:100

RANDOM

double RANDOM()

**Definition:** random.cpp:33

Seed

int Seed(int seed)

**Definition:** random.cpp:79

AskSeed

void AskSeed()

**Definition:** random.cpp:109


---

Generated on Thu Aug 14 2014 22:04:01 for Tissue Simulation Toolkit by  

 1.8.6
